# Systematic review of the diagnostic value of hydrops MRI in relation to audiovestibular function tests (electrocochleography, cervical vestibular evoked myogenic potential and caloric test)

**DOI:** 10.1007/s00405-022-07702-2

**Published:** 2022-10-27

**Authors:** Kumiko Yukawa Orimoto, Maria Vartanyan, Stephen J. O’Leary

**Affiliations:** 1 The University of Melbourne, The Royal Victorian Eye and Ear Hospital, 32 Gisborne St, East Melbourne, VIC 3002 Australia; 2 The Royal Victorian Eye and Ear Hospital, East Melbourne, 3002 Australia

**Keywords:** Electrocochleography, CVEMP, Caloric test, Meniere’s disease, Endolympatic hydrops, MRI

## Abstract

The objective of this systematic review is to compare the diagnostic value of endolymphatic hydrops (EH) magnetic resonance imaging (MRI) with audiovestibular function tests, including electro cochleography (ECochG), cervical vestibular evoked myogenic potential (cVEMP) and caloric tests for the diagnosis of definite Meniere’s disease (DMD). An electronic search was performed in the PubMed, Embase and Cochrane databases in August 2022. Original studies which reported the efficacy of gadolinium MRI for diagnosis of DMD were compared with ECochG, cVEMP and caloric tests from 2007 to 2022 published in English. Two reviewers extracted the methodology and results of MRI and functional tests, assessing them independently. A modified version of the Quality Assessment of Diagnostic Accuracy Studies (QUADAS-2) was used for the assessment of the quality and the risk of bias of each study. The proportion of DMD cases diagnosed by MRI hydrops vs corresponding functional tests were calculated and the relationship between MRI and functional tests were evaluated using the Cohen’s Kappa test. Concerning the MRI, the proportion diagnostic of DMD was 0.67 by cochlear EH and 0.80–0.82 by vestibular EH. Regarding the functional test, the propotiojn diagnostic of DMD was 0.48 by ECochG, 0.76 by cVEMP and 0.65 by caloric test. The findings of this systematic review were that the vestibular EH on imaging most effectively assisted in diagnosing DMD. Among the functional tests, cVEMP was the second most effective test. The agreement between imaging and cVEMP was moderate (0.44), indicating a gap between the patients identified by the imaging and functional tests based on the relatively small number of patients.

**Supplementary Information:**

The online version contains supplementary material available at 10.1007/s00405-022-07702-2.

## Introduction

Meniere’s Disease (MD) is characterized by spontaneous attacks of vertigo lasting from 20 min to 12 h with associated tinnitus, ear fullness, and fluctuating low frequency hearing loss in the affected ear before, during, or after an attack [1]. Its pathophysiology is still not completely clear, although it has been linked to increased hydraulic pressure (endolymph volume) within the inner ear, causing abnormal accumulation of endolymph fluid known as endolymphatic hydrops (EH) leading to episodic ear symptoms [2]. Temporal bone studies have shown that whilst all patients with MD symptoms show evidence of EH in at least one ear post-mortem, there are also patients with EH without signs or symptoms of MD [3]. This suggests that the presence of hydrops may not be an epiphenomenon but is rather a necessary condition of MD, it is, however, not enough to explain the mechanism of episodic attacks of vertigo and hearing loss in MD.

The diagnosis of MD predominantly relies on a complete history and physical examination according to the AAO–HNS criteria (1995) and the diagnostic criteria for MD formulated by the Committee of the Barany Society in 2015 [4]. As there is no gold standard test for the diagnosis of MD, it is only made when all other possible causes of the patient's symptoms have been ruled out [5]. It is sometimes difficult to differentiate MD from other conditions, especially migrainous vertigo [6] and benign recurrent vestibulopathy [7]. The symptoms can be variable, occurring over different time spans and the hearing loss may even recover before audiometric measurements are made. Patients with hearing loss and balance disorders are commonly diagnosed as MD, a misdiagnosis due to the lack of sufficiently sensitive and specific diagnostic tests [8, 9]. In about 20% of patients with MD, the vestibular and cochlear symptoms can take up to 5 years and in 10% of cases even more than 10 years to coincide resulting in diagnostic delay [5].

Because of these diagnostic challenges, the objective confirmatory test for the diagnosis and study of the pathophysiology of MD has been sought for a long time. Audiovestibular testing has been the hallmark of objective testing for many years but has in general failed to prove sufficiently sensitive. More recently, MR imaging of hydrops has become possible, with success in localising the labyrinthine region exhibiting hydrops. It is unclear which of these approaches provides the best indication of MD, and which should be preferred. This study has two objectives as below.

(1) To determine whether functional and imaging investigations are identifying the same or different groups of patients. We explored the inter-test agreement of identifying MD between a functional modality and the corresponding labyrinthine region imaged by hydrops MRI. This provided insights into the appropriateness of choosing and interpreting a particular test. The best tests may be those where there is highest agreement of MD across the testing modalities. (2) To provide guidance on the best end-organ to test for MD, whether functionally or by imaging. We reasoned that the best target for investigation (between cochlea, vestibule and semicircular canal) would identify the highest numbers of patients known to have MD, preferably across both functional and imaging modalities. We matched the tests based on the tested end-organ using electrocochleography (ECochG) vs cochlear hydrops; cervical vestibular evoked myogenic potential (cVEMP) vs vestibular hydrops; and caloric vs semicircular canal (SCC) or vestibular hydrops. The latter is premised on the assumption that advanced hydrops can herniate into the lateral SCC and impair the caloric response [10]. We did not choose pure tone audiometry as a cochlear site-specific functional test, because audiometry is already a requirement for the diagnosis of DMD. We also did not include video head impulse test (vHIT) in the analysis of the SCC functional testing, because there is a well-documented dissociation of normal vHIT and abnormal caloric test in MD [11].

In most studies, the visualization of the inner ear structures was enabled using a three-dimensional fluid-attenuated inversion recovery (3D-FLAIR) sequence following the intra-tympanic injection of gadolinium (IT-Gd) [12–16]. Intravenous Gd (IV-Gd) has become more popular recently, even though the contrast of the IT-Gd is more intense, as it allows the visualization of the bilateral endolymphatic spaces simultaneously without invading the tympanic membranes [17]. In addition, the waiting time for imaging is shorter with IV-Gd and it enables evaluation of the blood–labyrinthine barrier disruption. Previous studies reported that the positive rate of endolymphatic hydrops imaging (EHI) for definite MD (DMD) is around 80–95% in both IT and IV-Gd enhanced imaging [18–20]. On the other hand, the positive rate of EHI in healthy or non-MD ears was around 10% [21, 22].

There have been numerous studies regarding imaging methods, evaluation methods, and the pathophysiology of MD. The correlation between the occurrence and the degree of EHI and audiovestibular tests has also been rigorously investigated. Ziylan [23] assessed the diagnostic value of ECochG compared with MRI with IT-Gd administration in the systematic review. He concluded that there is a relatively low sensitivity and negative predictive value for click stimulus ECochG compared with MRI for detecting EH in patients with DMD. The location of the EH (either cochlea, vestibule or both), however, was not assessed. Therefore, we seek high level of evidence regarding the comparison of different end-organ diagnostic tests that would reflect a site-specific pathophysiological change.

## Materials and methods

This systematic review has been conducted with the following criteria recommended by the Preferred Reporting Items for Systematic Reviews and Meta-analyses (PRISMA-P).

### Selection criteria

Studies were selected according to the criteria outlined below. As there are no randomised controlled trials (RCTs), we included cross-sectional (CS), prospective and retrospective cohort studies of patients with DMD diagnosed by AAO–HNS criteria 1995 or Barany criteria 2015. We excluded case reports, case control studies, letters, abstracts, opinion papers and animal studies. Studies were selected in the time range 2007 to 2022, relevant articles were retrieved from their original publication (Online Appendix). Only articles published in English were included.

### Information sources and search strategies in August 2022

Information was sourced by an electronic search performed in the PubMed, Embase and Cochrane databases or by contact with the authors if applicable. Keywords used for the search included various synonyms of Gadolinium MRI and DMD with ECochG, cVEMP or caloric tests (Online Appendix). The search was updated toward the end of the review. PROSPERO was searched for ongoing or recently completed systematic reviews. As relevant studies were identified, reviewers checked for additional relevant cited articles. Two investigators (KO and MV) independently carried out data extraction and reviewed the full-text articles of all the retrieved trials of possible relevance. Disagreements were resolved by discussion.

### Quality assessment

The quality of each study and the risk of bias were assessed using the adjusted version of Quality Assessment of Diagnostic Accuracy Studies (QUADAS-2) tool. Two reviewers (KO and MV) evaluated all selected studies independently and conflicting judgements were resolved by discussion.

### Data extraction and synthesis

From each study the following information was extracted: first author, year of publication, study design, sample size of DMD, duration of MD, age range and different diagnostic test values and results. The latter includes the MRI specifications and each of the functional studies. For MRI, delivery methods of Gd, EH grading system, locations of EH and the rate of positive and negative EH scans were extracted. For functional tests, stimulus condition, cut off level and the rate of positive and negative tests were extracted. Study characteristics are presented in Tables 1, 2, 3, and 4. Inter-test agreement between MRI hydrops and each one of the functional tests was calculated using the Cohen’s kappa test. Some studies included other audiovestibular function tests, such as vHIT or oVEMP. We extracted only ECochG, cVEMP and Caloric test results which corresponded to the site-specific hydrops MRI.

**Table 1:** Characteristics of included studies

Paper	Year	Study design	Total (*n*)	DMD (*n*)	DMD criteria	Age (y)	Disease duration (y)	Gd contrast	Test
Yamamoto	2010	CS	24	8	AAO-HNS1995	38–66	0.083–8.5	IT	E
Fiorino	2011	CS	18	18	AAO-HNS1995	25–78	0.6–9	IT	E/V/C
Gurkov	2011	Prospective CS	37	37	AAO-HNS1995	24–77	0.25–25	IT	E/V/C
Fukuoka	2012	CS	20	20	AAO-HNS1995	22–80	NR	IT	E
Seo	2013	CS	26	26	AAO-HNS1995	28–65	NR	IT	E/V
Hornibrook	2015	Prospective CS	102	30	AAO-HNS1995	Ave 58 (all)	NR	IT	E
Katayama	2010	CS	40	19	AAO-HNS1995	Ave 53.7	NR	IT	V
Okumura	2016	CS	21	21	AAO-HNS1995	Med 62 (38–78)	NR	IV	V
Wesseler	2019	CS	27	14	Barany 2015	NR	NR	IT	V
Kato	2011	Prospective CS	24	13	AAO-HNS1995	29–69	0.42–10	IT	C
Fukushima	2019	CS	90	90	Barany 2015	13–86	NR	IT	C
Perez-Fernandez	2019	CS	22	22	Barany 2015	NR	NR	IT	C
Kitano	2020	CS	76	15	Barany 2015	38–66	0.5–5	IT	C
Paskoniene	2020	CS	105	55	Barany 2015	NR	NR	IV	C
Murofushi	2020	CS	14	8	Barany 2015	NR	NR	IV	V
Liu	2021	CS	69	69	Barany 2015	28–75	3.43 ± 2.9	IT	V/C
Oh	2021	CS	62	15	Barany 2015	44–78	5.2 ± 5.3	IV	V/C

*AAOHNS* American Academy of Otolaryngology, Head and Neck Surgery, *Ave* average, *C* caloric test, *CS* cross-sectional, *DMD* definite Meniere’s disease, *E* electrocochleography, *Gd* Gadolinium, *IT* intratympanic, *IV* intravenous, *Med* median, *NR* not reported, *V* cVEMP

**Table 2:** Conditions and results of MRI vs ECochG studies

Paper	MRI					ECochG						
	DMD pt (*n*)	MRI (*n*)	Cochlea + (*n*)	Cochlea − (*n*)	Cochlea hydrops positive criteria	Stimuli	Stimuli characteristics	ECochG (*n*)	ECochG + (*n*)	ECochG − (*n*)		Abnormal SP/AP
Yamamoto	8	8	7	1	Nakashima 2009	Click (ET)	Alternating click, rate 4/s, 500 times, BPF 100–3000 Hz	8	NR		NR	NTC
Fiorino	18	18	8	10	Normal, Reduced, Absent enhancement	Click (TT)	Alternating 0.1 ms click at 90 dB nHL, 200 times, BPF 10–3000 Hz	18	9		9	> 35%
Gurkov	37	34*	32	2	4-point Likert scale (0: no, 1: mild, 2: marked, 3: extreme hydrops)	Click (ET)	Alternating click at 90 dB nHL, rate 8.1/s, 1000 times, BPF 5–1500 Hz	28	NR		NR	NTC
Fukuoka	20	20	18	2	Semi-quantitative Positive/negative	Click (TT)	Alternating click at 100 dB nHL, rate 15/s, 1000 times	20	12		8	> 33%
Seo	26	26	21	5	Area EHI > area of scala vestibuli; hydrops ratio of > 50%	Click (TT) Tone burst (TT)	Click at 1 kHz Tone burst at 1 kHz	26	15 1		10	> 33% > 33%, |SP|≤ − 3 mV
Hornibrook	30	30	5	25	Nakashima 2009	Click (TT) Tone burst (TT)	Alternating 0.1 ms click at 90 dB nHL, rate 10/s Tone burst at 100 dB nHL, at 0.5, 1, 2, and 4 kHz	30 30	9 25		21 5	> 50% > 50%

*MRI excluded in 3 patients (see text)

*BPF* bandpass filter, *DMD* definite Meniere’s disease, *ECochG* electrocochleography*, ET* extratympanic, *NR* not reported, *NTC* no threshold criterion defined, *TT* transtympanic

**Table 3:** Conditions and results of MRI vs cVEMP studies

Paper	MRI					cVEMP				
	DMD pt (*n*)	MRI (*n*)	Vestibule + (*n*)	Vestibule − (*n*)	Vestibule hydrops positive criteria	Stimuli characteristics	cVEMP (*n*)	cVEMP + (*n*)	cVEMP − (*n*)	Abnormal cVEMP
Katayama	19 (21ears)	21	20	1	Nakashima 2009	5 Hz Click (105 dB)	21	17	4	Murofushi1996 Present/absent
Fiorino	18	18	11Vest/14 SCC	7	Normally enhancing, partially enhancing, non-enhancing	500 Hz Tone burst (95 dB nHL, 5/s, 128 repetitions)	18	9↓/3 absent	6	Amplitude decreased > 30% or absent
Gurkov	37	34*	26	8	0–4 grading	500 Hz Tone burst (105 dB SPL, 7 ms duration, 5/s)	27	NR	NR	IAR < 0.5
Seo	26	26	18	8	EHI > 50%	250 Hz Tone burst (95 dB HL, 5/s)	26	25	1	ASI > 22.8%
Okumura	21	20**	14	6	Vest 33% void of signal	500 Hz Tone burst (110 dB SPL, 5/s)	21	11	10	AR > 32.7%
Wesseler	14	14	NR	NR	Barath grading	500 Hz Tone burst (100 dB nHL)	7	6	1	AR > 35%
Murofushi	8	8	7	1	EHI > 50%	500 Hz, 1000 Hz Tone burst (125 dB SPL)	8	7	1	Tuning property test SLOPE < -19.9
Liu	69	65***	57	8	Bernaerts grading (none, slight, moderate, severe)	500 Hz Tone burst (95 dB nHL, 5/s)	48	NR	NR	AR > 40%
Oh	15	15	12	3	Nakashima 2009	500 Hz Tone burst (100 dB nHL)	15	3	12	AR $\ge$ 40%

*MRI excluded in 3 patients (see text), **MRI not available in 1 patient (see text), ***MRI excluded in 4 patients (see text)

*AR* asymmetry ratio, *ASI* asymmetry index of amplitude, *DMD* definite Meniere’s disease, *IAR* interaural amplitude ratio, *NR* not reported, *NTC* no threshold criterion defined, *SCC* semicircular canal, *Vest* vestibule

**Table 4:** Conditions and results of MRI vs Caloric studies

Paper	MRI						Caloric				
	DMD pt (*n*)	MRI (*n*)	Vestibule + (*n*)	SCC + (*n*)	Vestibule − (*n*)	Hydrops positive criteria	Stimuli	Caloric (*n*)	Caloric + (*n*)	Caloric − (*n*)	Abnormal caloric
Fiorino	18	18	11	8	7	C/V: normally enhancing, partially enhancing, non-enhancing	Water 30 °C, 44 °C, 200 ml, 30 s	18	14	4	CP > 25% (Jongkee)
Kato	13	13	13	NR	0	C/V: Nakashima 2009	Air 15 °C only, 6 L/min, 60 s	13	9	4	CP > 25% (cold only)
Gurkov	37	34*	26	NR	8	C/V: 0–3 Likert	Water 30 °C, 44 °C, 100 ml, 30 s	25	NR	NR	NTC
Fukushima	90	90	NR	NR	NR	%ROI/total—NTC	Water 30 °C 44 °C, 20 ml, 10 s	82	37	45	CP > 25% (Jongkee)
Perez-Fernandez	22	22	22	NR	0	Normal, mild, moderate, severe	Water 30 °C, 44 °C	22	12	10	CP > 25% (Jongkee)
Kitano	15	15	12	NR	3	C/V: Nakashima 2009	Water 20 °C 20 ml, 10 s	15	9	6	ENG max slow phase velocity < 10°/sec
Paskoniene	55	55	42	NR	13	Barath grade 1, 2	Water 30 °C, 44 °C, 30 s	55	32	23	CP > 25% (Jongkee)
Liu	69	65**	57	36	8	SCC: 4-tier grading system	Air 24 °C, 50 °C	65	38	27	CP > 25% (Jongkee)
Oh	15	15	12	NR	3	Nakashima 2009	Water 30 °C, 44 °C	15	8	7	CP > 35% (Jongkee)

*MRI excluded in 3 patients (see text), **MRI excluded in 4 patients (see text)

*CP* canal paresis, *C/V* cochlea/vestibule, *DMD* definite Meniere’s disease, *ENG* electronystagmography, *NR* not reported, *NTC* No threshold criterion defined, *ROI* Region of interest, *SCC* semicircular canal

## Results

### Data selection

**Fig. 1:**
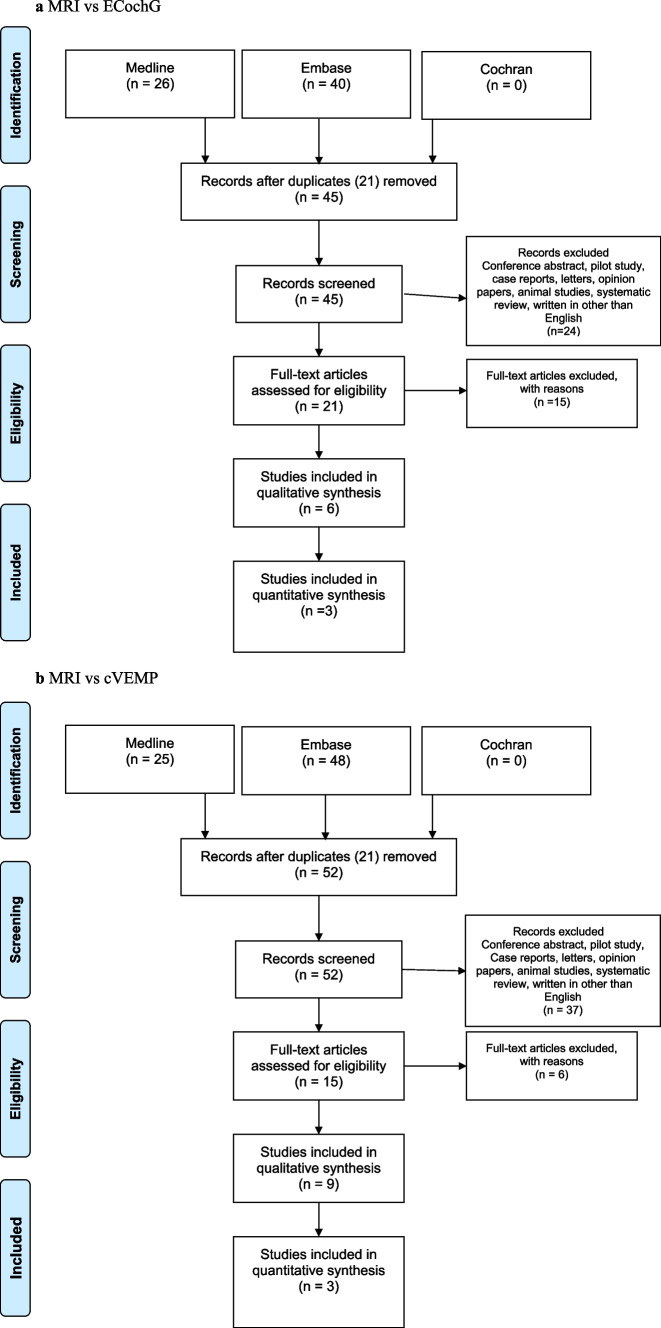

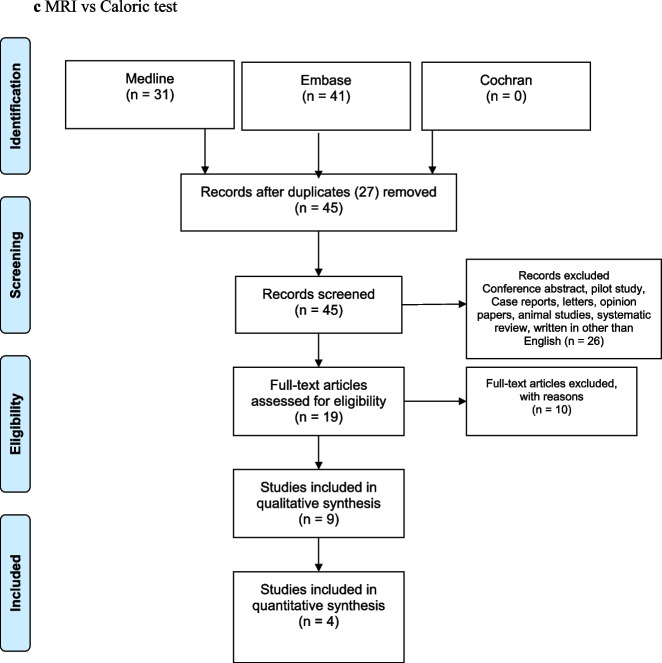
Search strategy flow chart. **a** MRI vs ECochG. **b** MRI vs cVEMP. **c** MRI vs Caloric test

Six CS studies (CSS) met the selection criteria for MRI vs ECochG review (PRISMA flow chart, Fig. 1a). Nine studies were chosen for MRI vs cVEMP review (PRISMA flow chart, Fig. 1b). The same three studies by Gurkov [16], Fiorino [19] and Seo [24] were included in the MRI vs ECochG and MRI vs cVEMP analysis as they assessed all those testing modalities. Nine studies were chosen for MRI vs Caloric review (PRISMA flow chart, Fig. 1c). The same studies by Gurkov [16] and Fiorino [19] were selected again as they looked into the MRI vs caloric relationship. The same studies from cVEMP by Liu [25] and Oh [26] were also selected in MRI vs cVEMP analysis. Table 1 shows the baseline characteristics of all the included studies.

### Quality assessment

Table 5 shows the QUADAS-2 method for included studies. A clinical diagnosis of DMD using the 1995 AAO–HNS criteria or 2015 Barany criteria [4] was considered as the reference test (gold standard) in the 6th question of this tool. The “Risk of bias” and “Applicability concerns” questions for studies by Yamamoto [27], Fukuoka [28], Seo [24], Hornibrook [29], Okumura [30], Katayama [31], Kato [32], and Kitano [33] were confirmed by contacting the authors.

**Table 5:** Critical appraisal

Paper	Risk of bias										Applicability concerns		
	Patient selection			Index test		Reference standard		Flow and timing			Patient selection	Index test	Reference standard
	Q1	Q2	Q3	Q4	Q5	Q6	Q7	Q8	Q9	Q10	Q11	Q12	Q13
Yamamoto	Y	Y	Y	Y	Y	Y	Y	Y	Y	Y	Y	Y	Y
Fiorino	NC	Y	Y	NC	Y	Y	Y	Y	Y	Y	Y	Y	Y
Gurkov	NC	Y	Y	NC	Y	Y	Y	Y	Y	N	Y	Y	Y
Fukuoka	Y	Y	Y	Y	Y	Y	Y	Y	Y	Y	Y	Y	Y
Seo	Y	Y	Y	Y	Y	Y	Y	Y	Y	Y	Y	Y	Y
Hornibrook	Y	Y	Y*	Y	Y	Y	Y	Y	Y	NC	Y	Y	Y
Katayama	Y	Y	Y	Y	Y	Y	Y	Y	Y	Y	Y	Y	Y
Okumura	Y	Y	Y**	Y	Y	Y	Y	Y	Y	N	Y	Y	Y
Wesseler	NC	Y	NC	NC	Y	Y	NC	NC	Y	N	Y	Y	Y
Kato	Y	Y	Y	Y	Y	Y	Y	Y	Y	Y	Y	Y	Y
Fukushima	Y	Y	Y	Y	N	Y	Y	Y	Y	Y	Y	Y	Y
Perez-Fernandez	Y	Y	Y	Y	Y	Y	Y	Y	Y	Y	Y	Y	Y
Kitano	Y	Y	Y	Y	Y	Y	Y	Y	Y	Y	N	Y	Y
Paskoniene	Y	Y	Y	Y	Y	Y	Y	Y	Y	Y	N	Y	Y
Murofushi	NC	Y	Y	NC	Y	Y	NC	NC	Y	Y	N	Y	Y
Liu	NC	Y	Y***	Y	Y	Y	Y	Y	Y	N	Y	Y	Y
Oh	NC	Y	Y	Y	NC	Y	Y	Y	Y	Y	N	Y	Y

Q1 Consecutive or random, Q2 Case control avoided, Q3 Avoid inappropriate exclusion, Q4 Results interpreted without knowing reference results, Q5 Prespecified threshold,

Q6 Likely to correctly classify the target condition, Q7 Results interpreted without knowing index results, Q8 Appropriate interval between index test and reference test,

Q9 All patients receive the same reference standard, Q10 All patients were included the analysis, Q11 All patients match the review question, Q12 Index test match the review question,

Q13 Reference test match the review question

*N* no, *NC* not clear, *Y* yes

*For further analysis reasons, inadequate gadolinium cases are classified as negative results

**One exclusion for renal failure

***Four exclusions as no gadolinium was seen in the any part of membranous labyrinth

### MRI cochlea vs ECochG

#### Description of studies included in MRI EH cochlea vs ECochG

All Hydrops MRI scans were performed in the 3D-FLAIR sequence with a 3-T MRI unit with IT-Gd. The EHI grading systems were not consistent across the studies. All studies assessed cochlea and vestibule separately. Yamamoto [27] and Hornibrook [29] adopted Nakashima’s 2009 scale (Table 6) [34]. Seo’s [24] index defined cochlear hydrops as present if the volume of scala media exceeded that of scala vestibuli. This is equivalent to Nakashima’s “significant” EHI level. Gurkov [16] used a 4-level Likert scale and Fiorino’s [19] scale defined 3 levels which reflected the extent of Gd uptake (enhancement) by the cochlea: normally enhancing (no hydrops), partially enhancing (some hydrops), and non-enhancing (extensive hydrops) (Table 2).

**Table 6:** Grading of endolymphatic hydrops using MRI [34]

Grade of hydrops	Vestibule (area ratio*)	Cochlea
None	≤ 33.3%	No displacement of Reissner’s membrane
Mild	> 33.3%, ≤ 50%	Displacement of Reissner’s membrane Area of cochlear duct ≤ area of the scala vestibuli
Significant	> 50%	Area of the cochlear duct exceeds the area of the scala vestibuli

^*^Ratio of the of the endolymphatic space to that of the fluid space (sum of the endolymphatic and perilymphatic spaces) in the vestibule measured on tracings of images

[34] Nakashima T, Naganawa S, Pyykko I, Gibson WP, Sone M, Nakata S, et al. Grading of endolymphatic hydrops using magnetic resonance imaging. Acta Otolaryngol Suppl 2009 (560):5–8

The clinical stage of MD varied between these investigations. Table 2 shows only the DMD cases that we extracted. Some MRI scan results were excluded from analysis in the original papers. Gurkov [16] performed MRI in a total of 37 cases, but only 34 were suitable for assessment, three were excluded from the analysis due to movement artefact, claustrophobia, and sub-clinical effusion in the middle ear. Hornibrook [29] found 10 out of 102 participants had inadequate Gd diffusion into the inner ear perilymph making the assessment of a hydropic status impossible. In another 30 cases the interpretation was too difficult, and therefore, only 61% (62/102) of patients who had good Gd entry were analysed. Out of these 30 cases of DMD, it is not clear how many cases did not have good enough Gd entry and they were excluded from his analysis. Other studies included all cases that had MRI performed. Fukuoka [28] evaluated the EH based on either unilateral or bilateral imaging in his study. When using unilateral MRI, EH was evaluated qualitatively from an image of the cochlea derived from 3D inversion recovery sequence utilizing a real reconstruction (3D real IR) image on the axial plane. In the case of bilateral MRI, a quantitative evaluation was performed using the ratio of affected to unaffected area < 0.90 as an indicator of EH from the multiplanar reconstruction.

To record ECochG, all authors except Yamamoto [27] and Gurkov [16] used trans-tympanic needle electrodes. The latter studies made extra-tympanic recordings with a silver ball electrode placed upon the tympanic membrane. Click stimuli were adopted in all cases. Hornibrook [29] and Seo [24] also delivered tone burst stimuli. The specifics of the click and tone burst are shown in Table 2.

#### Analysis

Yamamoto [27] showed that 7 out of 8 DMD cases had MRI EH in the cochlea. There was no mention of the cut off level for an abnormal SP/AP ratio, but these 7 EH cases had 38–83% of SP/AP ratio and the one non-EH case had 29% of SP/AP ratio. Authors concluded the SP/AP ratio in patients with significant MRI EH in the cochlea was more elevated than the SP/AP ratio in patients with none and mild EH (*p* < 0.05).

Fukuoka [28] reported positive ECochG in 12 out of 20 cases and positive EH in the cochlea in 18 out of 20 cases by evaluating unilateral MRI; this difference in positive rate was statistically significant (*p* < 0.05). SP/AP ratio > 33% was considered abnormal.

Gurkov [16] demonstrated cochlear MRI EH in 32 out of 37 cases. During ECochG, interpretable waveforms were obtained in 28 cases. There was no quantitative criterion of the abnormal SP/AP ratio mentioned for MD diagnosis and instead authors compared the grade of cochlear hydrops (*x*-axis, grade 0–3) to the value of the SP/AP ratio (*y*-axis). There was no clear correlation observed in these 28 cases (Spearman’s ρ = 0.27, *p* = 0.29).

In Fiorino’s study [19], all 18 cases of DMD showed a variable degree of impaired uptake of the inner ear. The cochlea showed EH in 8 cases. This study quantified the definition of an abnormal SP/AP ratio (> 35%). Increased SP/AP ratio was seen in nine cases, normal in six cases and absent in three cases. The conclusion was that there was no significant correlation between the extension of MRI EH and ECochG findings (ρ = 0.252, *p* = 0.308).

Seo [24] found that 21 out of 26 DMD cases had positive MRI findings of EH in the cochlea. Abnormal responses in ECochG occurred in 16 out of 26 (62%) of DMD cases. These cases demonstrated SP/AP elevations > 0.33 with click stimuli in 15 patients and SP ≤ − 3 µV with tone burst in one patient. Authors concluded that the differences in ECochG between the groups with MRI visualized and non-visualized cochlear EH were significant (*p* = 0.001).

Out of Hornibrook’s [29] 30 DMD cases, 14 patients had MRI EH (47%) and of these, 5 out of 14 had EH in the cochlea. On the other hand, ECochG showed an elevated SP/AP ratio (SP/AP ratio > 50% mentioned as abnormal) in 9 cases with click stimuli and in 25 cases with tone burst stimuli amongst 30 DMD cases. Author concluded that the use of tone burst ECochG increases the sensitivity of the test compared to click ECochG and that these techniques are more sensitive than Gd MRI scanning.

**Fig. 2:**
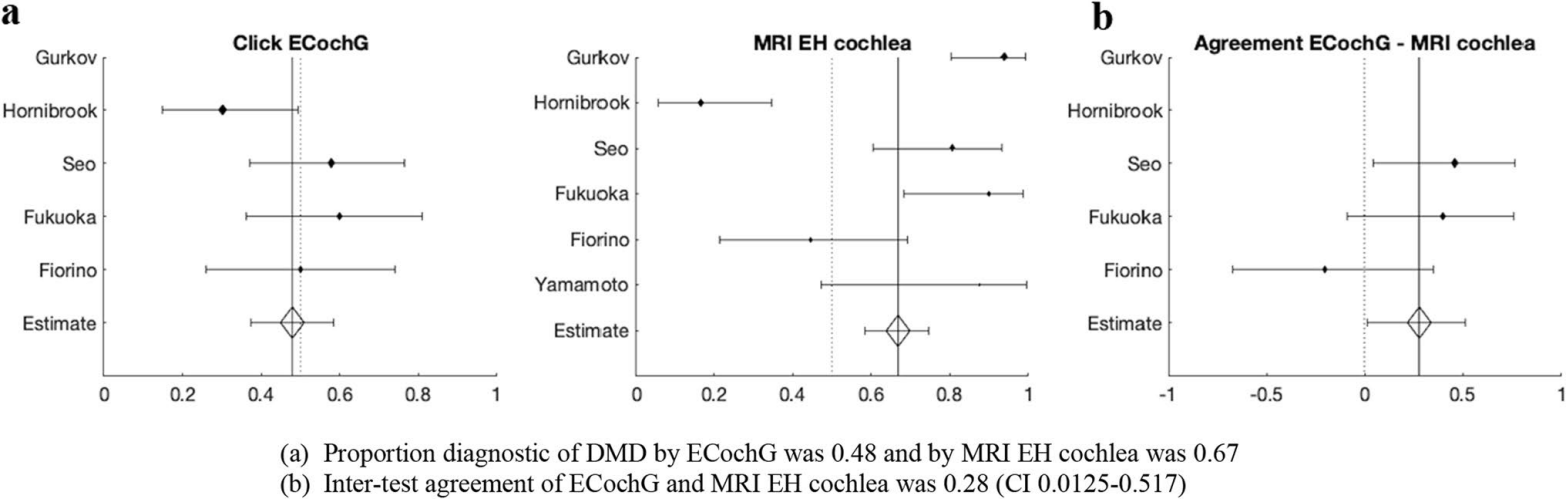
Relationship between MRI endolymphatic hydrops (EH) in the cochlea and click ECochG diagnostic of definite Ménière's disease (DMD)

Using ECochG with click stimuli, Gurkov [16], Fiorino [19], Fukuoka [28], Yamamoto [27] and Seo [24] found that there was no correlation between an elevated SP/AP ratio and MRI EH in the cochlea. Figure 2a shows that the proportion of cases diagnostic of DMD by ECochG was 0.48 and that of by MRI EH cochlea was 0.67. From these values, Cohen’s kappa was calculated for three studies, which specified both the number of MRI EH cochlea and ECochG positive tests, as well as both the number of MRI EH cochlea and ECochG negative tests. Cohen’s kappa between ECochG and MRI EH cochlea was 0.28 (CI 0.0125–0.517), which indicates a fair inter-test agreement (Fig. 2b).

### MRI vestibule vs cVEMP

#### Description of studies included in MRI EH vestibule vs cVEMP

Table 3 shows all DMD cases included in MRI EH vestibule vs cVEMP analysis. Regarding the grading of vestibule hydrops, Katayama [31], Okumura [30], Oh [26] and Murofushi [35] adopted Nakashima’s 2009 scale (Table 6) [34]. However, Okumura [30] judged vestibular hydrops positive when more than 33.3% of the vestibule was occupied by a low signal, which is equivalent to “mild’’ on Nakashima’s scale. Murofushi [35] considered vestibular hydrops positive when the area of the endolymphatic space was more than 50% of the vestibular fluid space, which is equivalent to “severe” on Nakashima’s scale. Fiorino [19] classified the vestibule as normally enhancing, partially enhancing, and non-enhancing. Gurkov [16] graded vestibular hydrops with the same 4-point Likert scale. Seo [24] graded EHI determined by the ratio of the area of endolymphatic space to the vestibular fluid space. Patients with no hydrops had half or less of that ratio, and those with certain hydrops had a ratio of more than 50%. The latter is equivalent to Nakashima’s significant hydrops. Wesseler [36] graded mild EHI and significant EHI by measuring the volume of the vestibule based on the histopathology of the temporal bones of healthy people and those of MD patients, taking into consideration Barath [37] and Pyykko’s [38] MRI grading system. In some DMD cases, MRI scans were not available. Okumura [30] performed MRI in 20 out of 21 patients. One could not be examined because of renal failure. As mentioned before Gurkov’s study [16] excluded 3 cases. Although Liu [25] performed MRI for all 69 cases, 4 cases were removed because of poor permeability of Gd contrast.

Regarding cVEMP testing, Katayama [31] used click stimuli, whereas all other authors used tone burst. Each of the authors adopted different criteria for cVEMP abnormality. All studies except Katayama [31] and Murofushi [35] used an abnormal interaural amplitude asymmetry ratio (AR) [39]. Cut-off levels in each study were heterogenous. Fiorino [19] used a difference greater than 30% as abnormal. Okumura [30] considered that AR at 110 dB SPL > 32.7% was abnormal. Seo [24] measured the latency of p13 and n23 and the amplitude between p13 and n23, in addition to asymmetry index of the amplitude (IAS). For this setup 22.8 ± 0.84% was normal. Wesseler [36] analysed the p13 and n23 latencies and AR. An AR of more than or equal to 35% was rated as abnormal. Liu [25] considered cVEMP AR of more than 40% to be abnormal. In Oh’s study [26], an AR equal to or more than 40% was considered abnormal. Gurkov [16] calculated the ratio of the absolute value of p13 and n23 of the affected and unaffected ears as interaural amplitude ratio. The author considered an interaural amplitude ratio of < 0.5 as pathological. As Gurkov’s interaural amplitude ratio (IAR) was an absolute value, this < 0.5 corresponded to > 33.3% of the others’ AR value. Katayama [31] reported that when cVEMP was within the noise level, it was judged as cVEMP absent. Murofushi [35] calculated cVEMP slope (SLOPE) with a SLOPE of less than -19.9 considered to indicate hydrops in the vestibule.

#### Analysis

Katayama [31] reported that MRI hydrops was observed in both the cochlea and the vestibule in all cases except one (only cochlea hydrops). CVEMP was absent in 17 out of 21ears.

In Fiorino’s study [19], 11 out of 18 cases showed partially or totally impaired Gd uptake in the vestibule. In these 11 cases, 9 showed a cVEMP abnormality. A cVEMP abnormality was observed in 12 out of 18, in 3 out of 12 cases it was absent, nine out of 12 cases showed decreased amplitude and 6 cases were normal. The total cVEMP abnormality rate of 67% (12/18) was higher than usually reported in MD. The author found that hydrops of all inner ear sites on imaging correlated with a reduced cVEMP response (ρ = 0.673, *p* = 0.004).

Gurkov [16] reported that vestibular hydrops was positive in 26 out of 34 MRI cases. CVEMP waveforms were reproducibly obtained in 27 patients. Gurkov concluded that the greater the degree of vestibular hydrops seen on MRI, the lower the Interaural amplitude ratio was on cVEMP (*n* = 27). This correlation (ρ = − 0.44) was significant (*p* < 0.05) and the cVEMP abnormality rate was 73%, higher than usually reported. As individual cases were not reported, it is not clear how many MRI positive cases were also cVEMP positive.

Seo [24] reported that out of 26 patients with DMD, 18 cases had MRI hydrops in the vestibule. On the other hand, 25 (96%) patients had abnormal ASI. In 18 patients with positive MRI, 17 had the absence of cVEMP responses and only 1 patient had a normal cVEMP response in an affected ear.

Okumura [30] reported MRI hydrops in the vestibule in 14 out of 20 cases (1case had no data because of renal failure), and cVEMP was abnormal in 11 out of 21 cases. Ten patients had hydrops-positive imaging and abnormal cVEMP. Wesseler [36] reported all 14 DMD patients showed hydrops, either cochlea or vestibule. CVEMP was only performed for 7 cases and 6 out of 7 were abnormal.

Liu [25] reported that 57 out of 65 DMD patients had some evidence of MRI hydrops in the vestibule. CVEMP was elicited in 48 of 65 (73.8%) patients. The author did not mention how many patients with MRI signs of hydrops in the vestibule had abnormal cVEMP, but they concluded that MRI hydrops in the vestibule significantly correlated with the asymmetry ratio of cVEMP (ρ = 0.407, *p* = 0.001).

In Oh’s study[26], 12 out of 15 DMD patients had MRI sign of hydrops in vestibule and 3 out of 15 patients had abnormal asymmetry ratio.

Murofushi [35] found 7 out of 8 DMD patients had MRI signs of hydrops in the vestibule and 7 out of 8 patients had cVEMP amplitude-growth slopes consistent with hydrops.

**Fig. 3:**
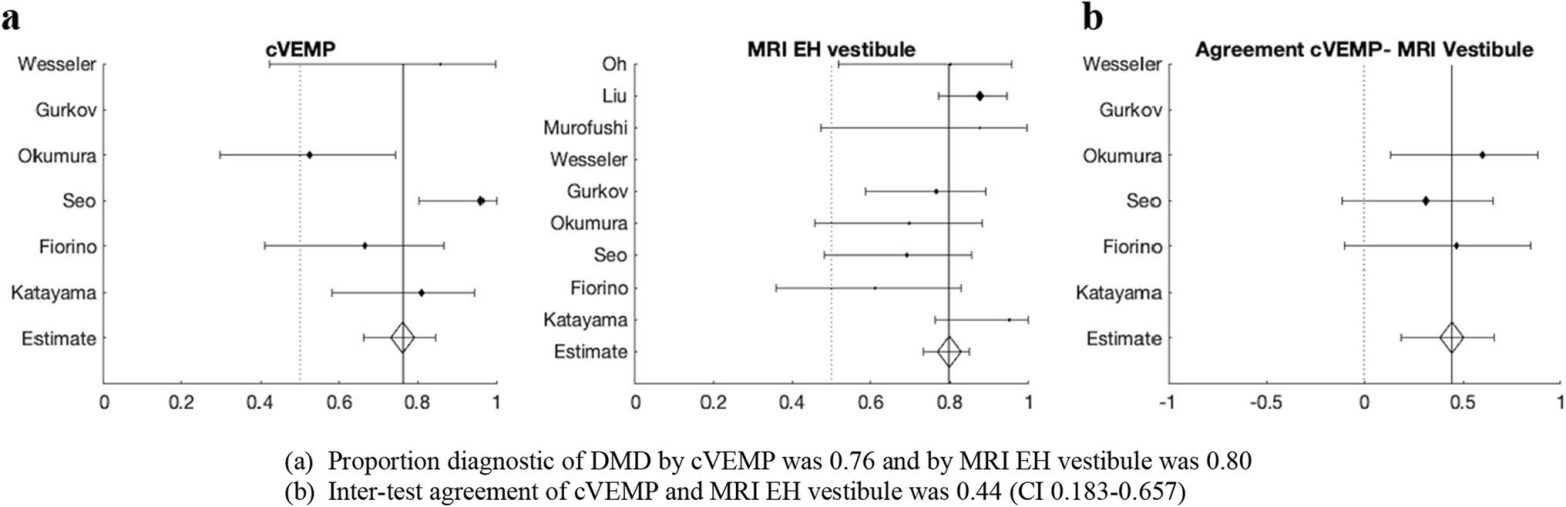
Relationship between MRI endolymphatic hydrops (EH) in the vestibule and cVEMP diagnostic of definite Meniere’s disease (DMD)

Figure 3a shows that the proportion of cases diagnostic of DMD by cVEMP was 0.76 and that by MRI EH in the vestibule was 0.80. Cohen’s kappa could be calculated for three studies, which have the number of both MRI and cVEMP positive tests and the number of both MRI and cVEMP negative tests. Kappa was 0.44, which indicates a moderate intertest agreement (CI 0.183–0.657) (Fig. 3b).

### MRI vestibule/SCC vs caloric test

#### Description of studies included in MRI EH vestibule/SCC vs caloric test

Table 4 shows all DMD cases included in this MRI EH vestibule/SCC vs caloric analysis. All studies except Perez-Fernandez [40] adopted 3D-FLAIR sequences for MRI. Perez-Fernandez [40] adopted 2D-FLAIR, 3D-FLAIR and 3D-IR. Regarding the grading of hydrops, Fiorino [19] and Liu [25] evaluated the semicircular canals (SCCs). Fiorino [19] judged all three canals as normally enhancing, reduced-enhancing, and non-enhancing. Liu [25] assessed lateral SCC, defined Grade 0 as no hydrops (a small visible herniation which was 1/3 less than the SCC with perilymph surrounding), Grade1(a larger herniation more than 1/3), Grade 2 (total invisibility of crura, which often accompanies stenosis of the canals) and Grade 3 (all SCCs were invisible).

The other papers used hydrops in the vestibule for the comparison with a caloric test. Kato [32], Kitano [33] and Oh [26] adopted Nakashima’s 2009 scale (Table 6) [34], Kato [32] also evaluated the degree of EH in the lateral SCC ampulla quantitatively. Kitano [33] assessed the vestibular herniation into the SCCs defined as EH herniation positive. Gurkov [16] adopted the same 4-point Likert scale as for the cochlea. Fukushima [41] measured total number of all pixels in the region of interest (ROI) and the number of pixels with negative signal intensity values, which represents vestibular EH in the ROI. The volume ratio of vestibular EH (vEH%) was calculated based on the ratio defined as the number of negative pixels for EH in the ROI divided by the total number of pixels in the ROI. There was no abnormal cut-off level specified. Perez-Fernandez [40] classified the vestibular EH as mild (sacculus and utricle individually seen), moderate (sacculus and utricles are fused with perilymph rim) and severe (completely occupied with endolymph). Paskoniene [42] categorized vestibular EH as Grade 1 (mild) and Grade 2 (significant) by Barath’s [37] grading system.

Regarding caloric stimulation, Fiorino [19], Gurkov [16] Fukushima [41] Perez-Fernandez [40], Paskoniene [42] and Oh [26] used the irrigation with various volumes of water at 30 and 44 °C and the time of stimulation as described in Table 4. These six studies measured the maximum slow-phase velocity (max-SPV) of nystagmus and the percentage of canal paresis (CP%) was calculated using Jongkees’ formula, CP > 25% was considered as abnormal in all papers except Gurkov [16] and Oh [26]. Oh [26] used > 35% as a cut off level and Gurkov [16] did not specify the cut-off level defining an abnormal response. Kato [32] used cool air stimulation (15 °C, 6 L/min, 60 s) and calculated CP by SPV values by the following formula: (unaffected—affected)/(unaffected + affected) × 100%, CP > 25% was considered abnormal. Kitano [33] used 20 °C water and measured SPV classifying CP as positive when SPV was < 10/s. Liu [25] used air stimulation with 24 °C and 50 °C and CP > 25% was considered abnormal.

#### Analysis

Although Fiorino [19] measured MRI hydrops in the SCCs and 8 out of 18 scans revealed EH in the lateral SCC, the statistical analysis was done with between the vestibular EH site (11out of 18 scans). Canal paresis “diagnostic” of EH (> 25%) was found in 14 of 18 cases, and the paresis value (percentage) was not significantly correlated with the extent of the hydrops (ρ = 0.403, *p* = 0.07). In Kato’s study [32], all 13 cases had positive EH in the vestibule on MRI and canal paresis was present in 9 out of 13 cases. An analysis of the degree of MRI EH in the ampullar region of the lateral SCC and the presence of canal paresis was not significant (*p* > 0.05 by Mann–Whitney *U* test).

Gurkov [16] found EH in the vestibule on MRI in 26 out of 34 cases as mentioned before. A caloric test was obtained in 25 patients, and the canal paresis was determined as a percentage. These measurements were not correlated significantly (ρ = 0.18, *p* = 0.29).

Fukushima [41] mentioned that the percentage of EH in the vestibule on MRI ranged widely from 3.2% to 64.1%. The median was 18.1% in a total of 90 patients. With the caloric tests, 37 out of 82 patients (45.1%) had canal paresis diagnostic of EH. The mean value of vEH% was 22.1 ± 2.2% for those with canal paresis diagnostic of EH (> 25%) and 16.8 ± 1.3% in those with lower canal paresis of values (*p* = 0.0467).

Perez-Fernandez [40] reported that all 22 DMD patients were vEH positive on MRI. As Perez-Fernandez [40] adopted 2D-FLAIR, 3D-FLAIR and 3D-IR, more than one sequence was performed on 18 out of 22 patients. The results for hydrops evaluation were concordant in all cases. CP exceeded 25% in 12 out of 22 cases, and these patients were more likely to be vEH positive on MRI (*χ*^2^; *p* = 0.028).

Kitano [33] found vEH positivity on MRI in 12 out of 15 DMD patients, vestibule herniation to the SCC was present in 9 out of 15 (lateral SCC herniation in the non-ampullated side and posterior SCC herniation in the common crus side). These nine cases were all CP positive (i.e., values exceeding 25%).

Paskoniene [42] found that 50 out of 55 cases had abnormal MRI; 42 of them were vEH positive and 8 had abnormalities other than hydrops. Thirty two out of 55 patients had a canal paresis diagnostic of EH.

Liu [25] assessed ampullar hydrops of the lateral SCC and 36 out of 65 had some grade of hydrops. A high canal paresis values (> 25%) diagnostic of EH were observed in 38 of 65 (58.5%) patients. Canal paresis diagnostic of EH (> 25%) was significantly correlated with lateral SCC hydrops (ρ = 0.367, *p* < 0.003). In Oh’s study [26], 12 out of 15 DMD patients had vestibular hydrops and 8 out of 15 patients had CP.

**Fig. 4:**
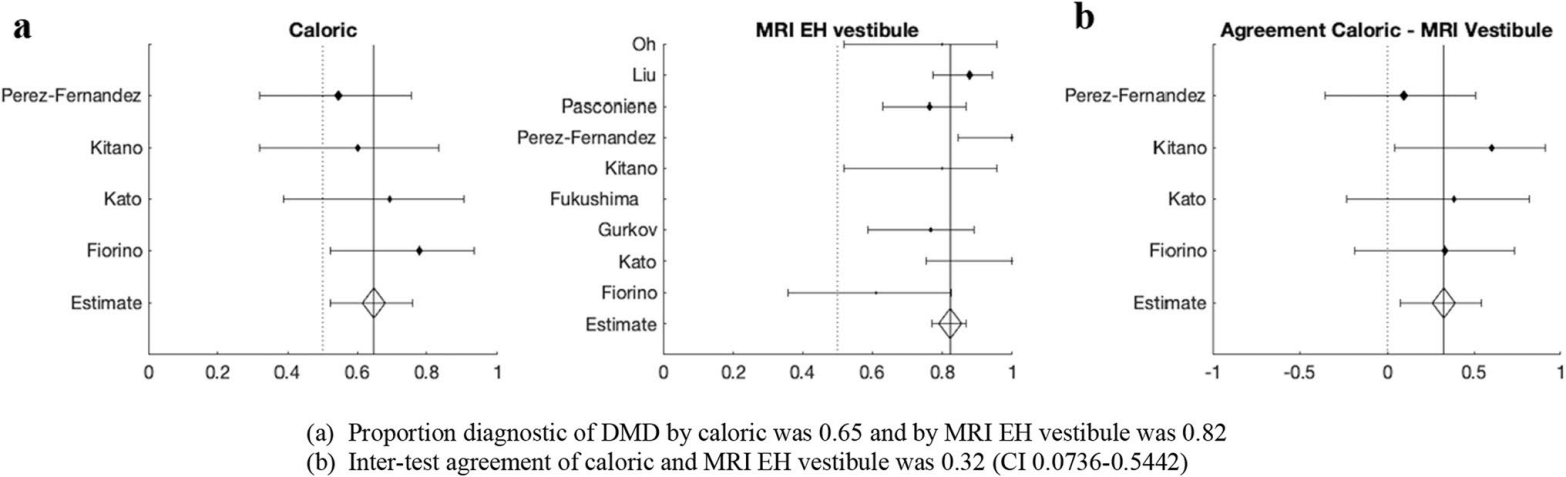
Relationship between MRI endolymphatic hydrops (EH) in the vestibule and caloric diagnostic of definite Meniere’s disease (DMD)

Figure 4a shows that the proportion of cases diagnostic of DMD by caloric test was 0.65 and that by MRI in the EH vestibule was 0.82. Cohen’s kappa was also calculated for four studies, incorporating the numbers of positive and negative MRI and caloric findings. Kappa was 0.32, which points to a fair inter-test agreement (CI 0.0736–0.5442) (Fig. 4b).

## Discussion

The main finding of this review is that EH on imaging of the vestibule more consistently supported the diagnosis of MD than any other test. Next best test was a cVEMP, in which the functional assessment of the saccular (vestibule) function more often supported EH than any other functional test. With the cVEMP, hypofunction was associated with an MD diagnosis, possibly indicating the predominance of more advanced MD in the studies extracted by us. However, the agreement between imaging and cVEMP was moderate (0.44), indicating that there was some disparity in the patients identified by each diagnostic test.

Several reasons may be proposed to explain why neither functional testing for cochlear EH (ECochG) nor the demonstration of EH on imaging of the cochlea supported an DMD diagnosis as strongly as vestibular imaging or functional assessment. This may be because of the considerable variability in the diagnostic criteria (SP/AP ratio cut-off) applied to ECochG in the different studies. Alternatively, it may be because the distention of the endolymphatic space and hair-cell biasing may not be well-correlated. This has been suggested previously [43], and it is likely to be the explanation for evidence by MRI of cochlear hydrops in asymptomatic patients who have never been diagnosed with MD, as well as for hydrops in the temporal bones of asymptomatic individuals [44]. According to this view, hair cell stereocilia will only be biased if there is sufficient pressure in the endolymphatic space to bias the basilar membrane. This may happen early in MD when cochlear hydrops has yet to be seen by MRI and during acute exacerbations with return to normal anatomy between the episodes. However, if the hydrops is chronic, Reissner’s Membrane may stretch and accommodate the increased endolymphatic volume. This will lower the pressure, and potentially decrease the membrane biasing. By these mechanisms, hydrops, which causes the radiological signs, and hair-cell biasing, that causes the both the auditory symptoms of MD and the SP/AP ratio to increase, may become disassociated. Early in the disease, cochlear EH may be less evident on imaging, but the SP/AP ratio is more likely to be increased. Late in the disease there is chronic hydrops; hair cells become dysfunctional causing an elevation of audiometric thresholds. The SP/AP ratio will be less prone to elevation but cochlear EH will be more evident on imaging. We analysed DMD cases only, which by diagnostic criteria requires an established cochlear dysfunction demonstrated by impaired hearing level, i.e., relatively advanced MD. This could have led to some selection bias.

Similarly, in the vestibular system, early in MD saccular function is prone to hyperfunction from biasing of the hair-cell stereocilia, but late in MD hair cell dysfunction causes hypofunction. Far advanced EH, as evidenced by herniation of the saccule into the lateral SCC, leads to permanent morphological changes of the sensory organs and consists with reduced or absent cVEMP and CP [45, 46].

As described above, an understanding that morphological EH and sensory function may become disassociated, and that test outcomes may vary according to the stage of disease, can help us appreciate why there is relatively low agreement between the imaging and functional testing of MD. This has important implications for choosing and interpreting diagnostic tests for this disease. Radiological imaging of the vestibule will more likely be diagnostic during later stages of MD. We would speculate that in the early stage of MD, hydrops MRI may be less useful. CVEMP asymmetry requires careful consideration of the disease stage; hypofunction in the affected ear provides reasonable support for late-stage MD, but it is unlikely to help with the diagnosis of early MD, where no change or hyperfunction may be seen. The caloric should only be considered to support a diagnostic of advanced-staged MD, where this diagnosis is supported by caloric hypofunction in the presence of an intact vHIT [47]. Other tests, such as the SP/AP ratio, or imaging of the cochlea may be supportive of a MD diagnosis but should not be relied upon.

## Limitations

Our study has several limitations. Firstly, the number of patients included in the studies was relatively small and was a highly selected population. In particular, the small number of patients might have lessened the significance of the relation between lesions affecting hydrops and function tests. Secondary, the heterogeneity of the studies including the stage of DMD, different methodologies of function tests and MRI conditions all of which made it difficult to generalize our findings to the broader population of patients with DMD presenting within a clinical setting. Further study comparing audiovestibular tests and MRI in a larger number of patients in a longitudinal setting will be needed. Thirdly, because the structural MRI cannot fully evaluate the functional extent of a lesion, we cannot be certain that the cochlea and vestibule that have no hydrops on MRI were functional.

## Conclusions

1. The highest diagnostic value for DMD was hydrops MRI in the vestibule.
2. Between the ECochG, cVEMP and caloric tests, cVEMP was most commonly correlated with hydrops MRI in the vestibule.
3. The advanced stage of DMD in most cases will both dilate the endolymph in the cochlea and vestibule and affect the audiovestibular function, so in many cases MRI would be a supportive test when combined with functional tests.

## Supplementary Information

Below is the link to the electronic supplementary material.

Supplementary file1 (DOCX 16 KB)
