# Supplementary material for: Systematic review of the diagnostic value of hydrops MRI in relation to audiovestibular function tests (electrocochleography, cervical vestibular evoked myogenic potential and caloric test)
Source: Eur Arch Otorhinolaryngol. 2022 Oct 27;280(3):947–62. doi: 10.1007/s00405-022-07702-2 (PMC9899732; doi:10.1007/s00405-022-07702-2)
Supplement: Supplementary file 1 — Supplementary file1 (DOCX 16 KB) [file 405_2022_7702_MOESM1_ESM.docx]

Appendix

| Ovid MEDLINE(R) and Epub Ahead of Print, In-Process, In-Data-Review & Other Non-Indexed Citations and Daily <2007 to August 16, 2022> | | |
| --- | --- | --- |
| Items | Searches | Results |
| ECochG vs MRI | ((gadolinium or gd) and (Magnetic Resonance Imaging or MRI) and (ECochG or electrocochleography) and (Meniere’s or Meniere)).mp. [mp=title, book title, abstract, original title, name of substance word, subject heading word, floating sub-heading word, keyword heading word, organism supplementary concept word, protocol supplementary concept word, rare disease supplementary concept word, unique identifier, synonyms] | 26 |
| CVEMP vs MRI | ((gadolinium or gd) and (Magnetic Resonance Imaging or MRI) and (vestibular evoked myogenic potential or VEMP or CVEMP) and (Meniere or Meniere’s)).mp. [mp=title, book title, abstract, original title, name of substance word, subject heading word, floating sub-heading word, keyword heading word, organism supplementary concept word, protocol supplementary concept word, rare disease supplementary concept word, unique identifier, synonyms] | 25 |
| Caloric vs MRI | ((gadolinium or Gd) and (Magnetic Resonance Imaging or MRI) and caloric and (Meniere or Meniere’s)).mp. [mp=title, book title, abstract, original title, name of substance word, subject heading word, floating sub-heading word, keyword heading word, organism supplementary concept word, protocol supplementary concept word, rare disease supplementary concept word, unique identifier, synonyms] | 31 |

| Embase Classic + Embase <2007 to 2022 August 16> | | |
| --- | --- | --- |
| Items | Searches | Results |
| ECochg vs MRI | ((gadolinium or gd) and (Magnetic Resonance Imaging or MRI) and (electrocochleography or ECochG) and (Meniere or Meniere’s)).mp. [mp=title, abstract, heading word, drug trade name, original title, device manufacturer, drug manufacturer, device trade name, keyword heading word, floating subheading word, candidate term word] | 40 |
| CVEMP vs MRI | ((gadolinium or gd) and (Magnetic Resonance Imaging or MRI) and (vestibular evoked myogenic potential or VEMP or CVEMP) and (Meniere or Meniere’s)).mp. [mp=title, abstract, heading word, drug trade name, original title, device manufacturer, drug manufacturer, device trade name, keyword heading word, floating subheading word, candidate term word] | 48 |
| Caloric vs MRI | ((gadolinium or Gd) and (Magnetic Resonance Imaging or MRI) and caloric and (Meniere or Meniere’s)).mp. [mp=title, abstract, heading word, drug trade name, original title, device manufacturer, drug manufacturer, device trade name, keyword heading word, floating subheading word, candidate term word] | 41 |

Title: Systematic review of the diagnostic value of Hydrops MRI in relation to audiovestibular function tests (electrocochleography, cervical vestibular evoked myogenic potential and caloric test).

Journal name: European Archives of Oto-Rhino-Laryngology

Authors: Kumiko Yukawa Orimoto, Maria Vartanyan, Stephen John O’Leary

Affiliation: The University of Melbourne, The Royal Victorian Eye and Ear Hospital

Corresponding author: Kumiko Yukawa Orimoto, [Kumiko.Orimoto@eyeandear.org.au](mailto:Kumiko.Orimoto@eyeandear.org.au)
